# Persistent Chemotherapy-Induced Peripheral Neuropathy as a Chronic Cancer Pain Syndrome: Mechanisms, Therapeutic Limitations, and Future Directions

**DOI:** 10.3390/cancers18142330

**Published:** 2026-07-19

**Authors:** Reed Alexander Firestone, Jamin Morrison

**Affiliations:** 1 Department of Medicine, Thomas Jefferson University Hospital, Philadelphia, PA 19107, USA; 2 MD Anderson Cancer Center at Cooper, Cooper University Hospital, Cooper Medical School of Rowan University, Camden, NJ 08103, USA; morrison-jamin@cooperhealth.edu

**Keywords:** chemotherapy-induced peripheral neuropathy, cancer pain, neuropathic pain, survivorship, palliative care, neuroinflammation, mitochondrial dysfunction, chronic pain, biomarkers, precision medicine

## Abstract

**Background/Objectives:** Chemotherapy-induced peripheral neuropathy (CIPN) is a common, dose-limiting complication of cancer therapy and a major contributor to chronic neuropathic pain among cancer survivors. Although traditionally managed as a treatment-related toxicity, persistent CIPN frequently persists long after chemotherapy completion and is increasingly recognized as a major survivorship and palliative care challenge. This review synthesizes current mechanistic, clinical, and translational evidence and reframes persistent CIPN as a chronic neuropathic cancer pain syndrome. **Methods:** A narrative review of peer-reviewed literature was conducted using PubMed/MEDLINE, Embase, and the Cochrane Library, supplemented by manual review of key references and clinical guidelines. Emphasis was placed on mechanistic investigations, clinical management, survivorship, palliative care, and emerging therapeutic strategies relevant to CIPN. **Results:** Current evidence suggests that persistent CIPN reflects a multifactorial chronic neuropathic pain state involving neuronal injury, mitochondrial dysfunction, oxidative stress, neuroimmune activation, and maladaptive nociceptive signaling. Existing pharmacologic therapies provide limited symptomatic benefit and largely fail to address the underlying biologic mechanisms that contribute to symptom persistence. Emerging approaches involving mitochondrial-targeted therapies, neuroimmune modulation, biomarker-guided risk stratification, and multimodal supportive care suggest the potential for earlier intervention and disease modification rather than symptom palliation alone. **Conclusions:** Persistent CIPN should be conceptualized as a chronic neuropathic cancer pain syndrome rather than solely as a treatment-related toxicity. Future progress will likely require mechanism-informed therapeutic development, biologic stratification, earlier intervention, and survivorship-focused multidisciplinary care to improve long-term outcomes and quality of life in patients living with persistent CIPN.

## Simple Summary

Chemotherapy-induced peripheral neuropathy (CIPN) is a common and often persistent complication of cancer treatment that can cause chronic neuropathic pain, impaired mobility, sleep disturbance, and reduced quality of life. Although CIPN is traditionally managed as a treatment-related toxicity, many patients continue to experience symptoms long after completing chemotherapy, highlighting the need for improved long-term management strategies. In this review, we examine the biologic mechanisms underlying CIPN, discuss the limitations of current therapies, and explore emerging mechanism-informed and disease-modifying treatment approaches. We further argue that persistent CIPN should be conceptualized as a chronic cancer pain syndrome with important implications for survivorship, palliative care, and future therapeutic development.

## 1. Introduction

Chemotherapy-induced peripheral neuropathy (CIPN) is one of the most common and clinically consequential toxicities of systemic cancer therapy, affecting approximately 30–60% of patients overall and an even greater proportion of those receiving neurotoxic agents such as taxanes, platinum compounds, vinca alkaloids, and proteasome inhibitors [1,2,3,4,5]. Although CIPN often develops during active treatment, symptoms frequently persist long after chemotherapy completion, resulting in chronic pain, disability, and reduced quality of life among cancer survivors [6,7,8,9,10].

Clinically, CIPN is typically described as a distal symmetric sensory neuropathy characterized by numbness, tingling, burning pain, allodynia, and impaired proprioception [2,6,9,11,12]. Many patients also develop gait instability, impaired manual dexterity, weakness, and increased fall risk, leading to substantial functional impairment and loss of independence [3,4,9,13]. Beyond symptom burden, CIPN is often treatment-limiting, necessitating chemotherapy dose reductions, delays, or discontinuation that may compromise oncologic outcomes [2,6,14,15].

Despite its prevalence and clinical impact, CIPN remains conceptually underdeveloped in oncology practice. It is typically viewed as a treatment-related toxicity requiring monitoring to preserve chemotherapy delivery. However, this framework fails to capture the persistence of symptoms, the complexity of the underlying pain mechanisms, and the broad physical and psychosocial consequences that extend into survivorship. As survivorship improves, persistent CIPN increasingly behaves as a chronic neuropathic cancer pain syndrome requiring long-term multidisciplinary management [6,7,10,16]. Growing evidence suggests that persistent CIPN is maintained by interconnected biologic mechanisms that extend beyond acute neuronal injury and overlap with those implicated in other chronic neuropathic pain syndromes, supporting a model in which symptoms are actively maintained rather than simply reflecting residual nerve damage [10,17,18,19,20,21,22,23,24,25,26,27]. Importantly, the mechanisms responsible for initiating chemotherapy-induced nerve injury are not necessarily the same as those that sustain persistent neuropathic pain after chemotherapy cessation. Distinguishing these phases provides an important framework for understanding disease progression and developing mechanism-specific therapies.

This review synthesizes current mechanistic, clinical, and translational evidence supporting a broader framework in which persistent CIPN is conceptualized as a chronic neuropathic cancer pain syndrome rather than solely a treatment-related toxicity. We review the underlying biology of persistent CIPN, examine the limitations of current treatment strategies, and evaluate emerging disease-modifying approaches. Ultimately, we argue that persistent CIPN should be conceptualized as a chronic cancer pain syndrome, with important implications for survivorship, functional outcomes, multidisciplinary cancer care, and future therapeutic development.

## 2. Materials and Methods

We examine CIPN through the integrated perspectives of cancer pain, survivorship, palliative care, and emerging mechanism-informed therapeutics. Rather than systematically cataloging all published studies, the objective was to synthesize evidence most relevant to understanding persistent CIPN as a chronic neuropathic cancer pain syndrome.

This review was designed as a narrative review rather than a systematic review; therefore, no formal systematic review methodology (e.g., PRISMA) was employed.

Literature was identified through searches of PubMed/MEDLINE, Embase, and the Cochrane Library, supplemented by manual review of references from key publications, consensus statements, and clinical practice guidelines. Priority was given to studies addressing clinical management, chronic symptom burden, biologic mechanisms, survivorship outcomes, and therapeutic innovation. Recent literature was preferentially incorporated when evaluating evolving areas such as neuroimmune signaling, mitochondrial dysfunction, biomarker development, precision medicine, and disease-modifying interventions, while landmark studies were included to provide historical and conceptual context.

Evidence was synthesized according to clinical relevance, methodological rigor, translational applicability, and potential implications for long-term patient outcomes. Particular emphasis was placed on investigations of persistent neuropathic pain mechanisms, limitations of current symptom-directed therapies, survivorship-related functional impairment, and emerging disease-modifying strategies. This synthesis was intended to provide a clinically relevant framework linking mechanistic insights to future therapeutic development and multidisciplinary survivorship care.

## 3. Clinical Phenotype, Functional Consequences, and Assessment Challenges

### 3.1. Clinical Presentation

Although often described as a distal symmetric sensory neuropathy in a stocking–glove distribution, persistent CIPN is clinically heterogeneous, with substantial variation in symptom type, severity, pain phenotype, temporal evolution, and functional impairment across patients and chemotherapy classes. Patients may experience numbness, paresthesias, burning pain, electric shock-like sensations, cold hypersensitivity, or impaired tactile sensation, with the relative prominence of pain, sensory loss, and functional impairment varying considerably across individuals [23,28,29,30,31].

Motor manifestations, while less common than sensory symptoms, are clinically significant when present and may include weakness, impaired balance, gait instability, and deficits in dexterity, particularly in patients receiving vinca alkaloids or those with advanced cumulative neurotoxicity [29,32,33,34]. The temporal evolution of CIPN is similarly variable. Some neurotoxic agents produce acute symptom flares during active treatment, whereas others are associated with cumulative toxicity or delayed symptom progression following chemotherapy completion.

While this review focuses specifically on CIPN, clinicians should recognize that certain chemotherapeutic agents may also produce musculoskeletal pain syndromes, including arthralgias and myopathies, which may co-exist with neuropathic symptoms and require distinct diagnostic and therapeutic considerations [35,36,37,38].

This heterogeneity complicates both assessment and management, particularly when biologically distinct neuropathic syndromes are approached using a uniform clinical framework rather than recognition of agent-specific patterns and patient-specific trajectories.

### 3.2. Functional Consequences and Survivorship Burden

The clinical significance of CIPN lies not only in its symptoms but also in their downstream functional consequences. Patients with persistent neuropathy frequently report difficulty walking, maintaining balance, climbing stairs, driving, sleeping, or performing tasks requiring fine motor coordination [29,32,33,34]. These limitations may be particularly debilitating for older adults, individuals with pre-existing mobility impairment, and survivors attempting to return to work or regain independence following cancer treatment.

CIPN also exerts a substantial psychosocial burden. Chronic pain, sleep disruption, fear of falling, frustration with physical limitations, and the perceived invisibility of symptoms may contribute to anxiety, depressed mood, and reduced quality of life [20,39,40]. These effects often persist long after cancer treatment has concluded, complicating survivorship and increasing the need for multidisciplinary supportive care.

From an oncologic perspective, CIPN is additionally consequential because it frequently disrupts treatment delivery. Dose reductions and treatment discontinuations undertaken to limit neuropathy may preserve long-term neurologic function but can also compromise disease control in certain settings [23,28,41]. This tension between oncologic efficacy and chronic symptom burden represents one of the defining clinical dilemmas of CIPN.

### 3.3. Challenges in Assessment and Clinical Recognition

Despite its prevalence, CIPN remains inconsistently assessed in routine clinical practice. Patient-reported outcome measures such as the EORTC-CIPN20 and FACT/GOG-Ntx provide sensitive assessments of symptom burden and functional impairment but remain underutilized outside research settings [23,42,43]. In contrast, clinician-based tools such as CTCAE grading are more commonly used but may underestimate symptom severity, particularly in early or moderate disease [42].

A major limitation of clinician-based assessment is its emphasis on objective deficits over patient-reported symptoms. Pain, numbness, and altered sensation may therefore be underestimated when neurologic findings are absent or subtle. This is particularly relevant because many patients with severe pain do not demonstrate corresponding abnormalities on electrophysiologic testing, suggesting that mechanisms beyond structural nerve injury, including central sensitization and altered nociceptive processing, contribute to symptom burden [44].

Objective measures such as nerve conduction studies, quantitative sensory testing, and skin biopsy can improve diagnostic precision but are difficult to implement routinely in oncology practice. Emerging biomarkers, particularly serum neurofilament light chain (NfL), have shown promise as indicators of neuroaxonal injury. NfL levels increase during chemotherapy, correlate with neuropathy severity, and may predict future neurotoxicity [30,31,40]. In one study, NfL levels measured after two cycles of paclitaxel predicted subsequent grade 3 neuropathy with 80% sensitivity and 79% specificity [40]. However, proposed cutoff values vary across chemotherapy classes and require further validation. Other candidate biomarkers, including glial fibrillary acidic protein (GFAP), brain-derived neurotrophic factor (BDNF), circulating mitochondrial DNA, and microRNAs, remain under investigation [30,31,34,40].

Currently, no single assessment strategy is both practical and biologically informative. This gap may delay intervention and limit recognition of patients transitioning from acute neurotoxicity to persistent chronic neuropathic pain.

## 4. Biological Mechanisms of CIPN: From Nerve Injury to Chronic Pain

Acute chemotherapy exposure initiates peripheral nerve injury through mechanisms that differ among neurotoxic agents, including microtubule disruption, dorsal root ganglion toxicity, oxidative stress, and mitochondrial injury. Although these initiating events explain the development of acute neurotoxicity, they do not fully account for why neuropathic pain persists months or years after treatment. Increasing evidence suggests that persistent CIPN is maintained by interconnected biologic processes—including mitochondrial dysfunction, neuroimmune activation, peripheral and central sensitization and maladaptive neuroplasticity—that continue long after chemotherapy exposure has ended. Understanding this distinction between mechanisms of injury initiation and chronic symptom maintenance provides an important framework for developing disease-modifying therapies.

### 4.1. Mitochondrial Dysfunction and Oxidative Stress

Mitochondrial dysfunction is one of the most consistently implicated mechanisms in CIPN and may represent a common biologic denominator across multiple neurotoxic agents. Chemotherapy-induced mitochondrial injury reduces ATP generation, impairs calcium homeostasis, and increases oxidative stress, thereby creating a cellular environment prone to neuronal injury and failed recovery [17,18,19,20]. The “mitotoxicity theory” of CIPN proposes that the accumulation of dysfunctional mitochondria within sensory neurons drives axonal degeneration, neuronal sensitization, and maladaptive central nervous system changes that ultimately establish a chronic neuropathic pain state [19,45,46]. Peripheral sensory neurons are especially vulnerable to mitochondrial dysfunction because of their high metabolic demands and extensive axonal length, which may help explain both the predominance of distal sensory symptoms and the incomplete recovery observed in many patients [17,18,19,20,45,46].

Peroxynitrite-mediated inactivation of mitochondrial superoxide dismutase contributes to bioenergetic deficits in paclitaxel, oxaliplatin, and bortezomib models, while a peroxynitrite decomposition catalyst prevented mechano-hypersensitivity without compromising antitumor activity [19,20]. Paclitaxel has also been shown to induce mitochondrial fragmentation in human sensory-like neuron cells by downregulating fusion proteins (MFN1/MFN2) and upregulating the fission protein Drp1, while inhibition of excessive mitochondrial fission with the peptide inhibitor P110 prevented neurotoxicity [47]. Similarly, cisplatin depletes mitofusin-2 in peripheral nerves, causing mitochondrial damage and contributing to neuropathy through disrupted mitochondrial dynamics [48].

Recent work has expanded this mitochondrial model beyond simple oxidative damage to focus on mitochondrial quality control as a dynamic and therapeutically targetable process. Im et al. demonstrated that a novel isoquinoline mitophagy inducer (ALT001) ameliorated paclitaxel-induced peripheral neuropathy in both *Drosophila* and mouse models in a mitophagy-dependent manner, importantly without interfering with paclitaxel’s cytotoxic effects on cancer cells [49]. Zhao et al. showed that Parkin-mediated mitophagy is decreased in oxaliplatin-induced peripheral neuropathy and that pharmacologic restoration of mitophagy with salidroside alleviated nerve injury and pain [50]. Together, these findings move the field beyond descriptive statements regarding oxidative stress and toward more precise molecular pathways—including mitophagy regulation, mitochondrial dynamics, and metabolic-epigenetic crosstalk—that may support future disease-modifying therapeutic strategies [45,46,47,48,49,50].

At the same time, the translational pathway remains uncertain. Antioxidant and mitochondrial-targeted strategies have shown mixed or limited efficacy in clinical settings, suggesting either that mitochondrial dysfunction alone does not fully account for CIPN or that interventions must be delivered with greater biologic precision and at earlier stages of disease than current approaches allow [51,52]. Collectively, these findings suggest that mitochondrial dysfunction may contribute not only to the initial neurotoxic insult but also to the maintenance of persistent neuropathic pain after chemotherapy has ceased.

### 4.2. Neuroinflammation and Neuroimmune Crosstalk

Neuroinflammation is another major mechanistic axis in CIPN and likely plays a central role in both symptom persistence and pain amplification. Chemotherapy can activate macrophages, satellite glial cells, and microglia, resulting in the release of cytokines such as TNF-α, IL-1β, and IL-6. These inflammatory mediators sensitize peripheral and central nociceptive pathways and may contribute to the persistence of symptoms long after chemotherapy cessation [12,29,53].

Importantly, emerging evidence suggests that this inflammatory response is not simply diffuse but may instead be organized through specific neuroimmune signaling circuits. Jia et al. demonstrated that neutrophil extracellular traps (NET) accumulate along a gut–blood–dorsal root ganglion axis in CIPN models and that fucoidan reduced NET accumulation and neuropathic symptoms [54]. Yang et al. further showed that fucoidan may act through Gas6/MerTK signaling to suppress neuroinflammation [55]. Together, these findings suggest that neuroimmune dysregulation in CIPN may involve targetable pathways rather than generalized inflammation alone.

McKiver et al. identified astrocyte-elevated gene-1 in myeloid cells as a key driver of CIPN in preclinical models, further implicating innate immune signaling as a central contributor to disease biology [56]. Collectively, these studies support a model in which CIPN is sustained by active immune–neuronal communication rather than passive residual injury [53,54,55,56].

Supporting the translational relevance of these findings, Starkweather reported increased circulating IL-6 activity in breast cancer survivors with painful CIPN compared with those without painful neuropathy, suggesting that inflammatory signaling may also contribute to persistent neuropathic pain in humans [57].

Although most evidence remains predominantly pre-clinical, emerging clinical studies support the potential relevance of neuroimmune pathways in human CIPN, while emphasizing the need for prospective validation.

### 4.3. Axonal Injury, Dorsal Root Ganglion Toxicity, and Structural Damage

Axonal injury remains a foundational component of CIPN pathophysiology. Taxanes and vinca alkaloids disrupt microtubule dynamics, impair axonal transport, and promote distal axonal degeneration [58,59]. Platinum compounds accumulate in dorsal root ganglia and induce DNA damage, oxidative stress, and mitochondrial injury, contributing to neuronal toxicity and persistent sensory dysfunction [59,60].

The structural injury model remains clinically useful because it explains many of the observed features of CIPN, including the distal symmetric distribution of symptoms and the cumulative relationship between exposure and severity [58,59]. However, it is increasingly clear that structural injury alone does not fully explain chronic pain. Some patients with significant sensory loss have relatively little pain, whereas others experience severe pain despite limited measurable deficits. This mismatch suggests that structural injury is only one component of a broader disease process involving maladaptive nociceptive and neuroimmune signaling [44,61].

In practical terms, structural injury may be best viewed as the initiating event that triggers downstream mechanisms—including mitochondrial dysfunction, neuroinflammation, and neuronal sensitization—that ultimately shape whether patients recover, plateau, or transition into chronic neuropathic pain [17,18,19,20,53,54,55,56,57,58,59,60,61].

Major neurotoxic chemotherapy classes differ substantially in their mechanisms of neuronal injury, temporal patterns of toxicity, and clinical manifestations of CIPN (Table 1).

### 4.4. Nociceptor Sensitization, Ion Channels, and Pain Amplification

Persistent pain in CIPN also reflects maladaptive changes in neuronal signaling. Altered expression and function of voltage-gated sodium channels, calcium channels, and transient receptor potential (TRP) channels contribute to increased neuronal excitability, spontaneous activity, and ectopic firing [58,59,62]. These mechanisms are particularly relevant in patients whose symptoms are dominated by pain, cold hypersensitivity, or allodynia rather than numbness alone.

Kim et al. demonstrated upregulation of NGF/TrkA-related proteins in dorsal root ganglia in a paclitaxel-induced peripheral neuropathy model, implicating neurotrophin signaling in nociceptor sensitization [63]. This finding links structural injury with sustained pain signaling and suggests that chronic pain in CIPN represents an actively maintained biologic process rather than simply a residual consequence of nerve loss.

These observations align CIPN with other chronic neuropathic pain syndromes in which nociceptive amplification and central sensitization sustain symptoms long after the initial insult has resolved [60,61]. Beyond central sensitization, sustained nociceptive signaling may also induce maladaptive neuroplastic changes within the spinal cord and paraspinal pain-processing networks [61,62]. Experimental studies suggest that activation of dorsal horn neurons, spinal glial cells, and higher-order pain centers contributes to persistent pain amplification. In addition, impaired descending inhibitory pathways mediated by serotonergic and noradrenergic circuits may further perpetuate chronic pain, providing a potential mechanistic explanation for the modest efficacy of duloxetine in painful CIPN [17,23,61].

Together, these findings help explain why symptom severity often correlates poorly with objective measures of nerve injury and support a model in which CIPN evolves from an acute neurotoxic injury into a persistent pain state maintained through ongoing peripheral and central nervous system dysregulation.

**Figure 1:**
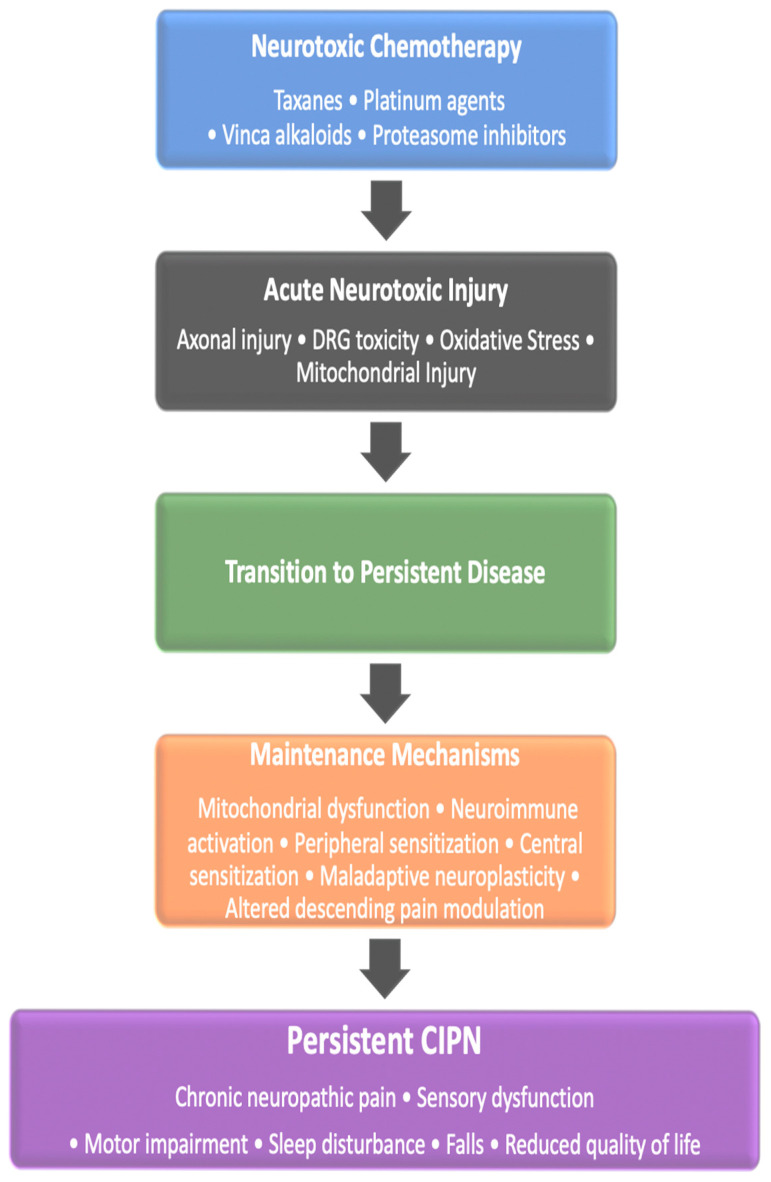
Proposed mechanistic framework for the development of persistent CIPN. Neurotoxic chemotherapy initiates acute peripheral nerve injury through agent-specific mechanisms. In susceptible individuals, persistent CIPN is maintained by interconnected biologic processes, resulting in chronic neuropathic pain, functional impairment, and reduced quality of life.

Collectively, these interconnected mechanisms support a model in which chemotherapy-induced neuronal injury initiates a cascade of mitochondrial dysfunction, neuroimmune activation, nociceptor sensitization, and central sensitization that culminates in persistent CIPN as a chronic neuropathic cancer pain syndrome (Figure 1).

## 5. Therapeutic Limitations and Current Management Challenges

Despite the substantial clinical burden of CIPN, therapeutic options remain limited and largely focused on symptom management rather than disease modification. Current pharmacologic and non-pharmacologic interventions provide variable benefit, and many patients continue to experience persistent pain, sensory dysfunction, and impaired quality of life. These limitations highlight important gaps in our current understanding of CIPN biology and underscore the need for more mechanism-informed therapeutic approaches.

### 5.1. Pharmacologic Management: Limited Efficacy and Persistent Gaps

Despite growing recognition of CIPN as a major survivorship and chronic pain issue, pharmacologic treatment options remain limited. Current therapies are directed primarily toward symptom management rather than reversal of the biologic processes that sustain neuropathy and chronic pain, and many patients continue to experience persistent symptoms despite treatment.

Among available therapies, duloxetine remains the only pharmacologic agent with moderate evidence supporting its use in established painful CIPN and is the only medication specifically recommended by ASCO guidelines [6,16,64]. Randomized trials have demonstrated modest reductions in pain severity, particularly in oxaliplatin-induced neuropathy, although responses remain variable and frequently incomplete [6,65]. In practice, adverse effects including fatigue, nausea, somnolence, and dizziness may further limit tolerability.

Other agents commonly used for neuropathic pain—including gabapentinoids, tricyclic antidepressants, topical therapies, and opioids—have shown inconsistent or limited efficacy in CIPN [16,65]. Gabapentin and pregabalin are frequently prescribed despite limited high-quality evidence of benefit [16,66]. Tricyclic antidepressants may provide symptomatic relief in selected patients but are often limited by anticholinergic effects, sedation, and cardiovascular toxicity, particularly in older adults and medically complex cancer survivors [16,65]. Opioids may transiently reduce pain severity but do not address the underlying neurobiology of CIPN and carry substantial risks related to sedation, respiratory depression, constipation, dependence, and impaired function [16,65,66].

The limited efficacy of current therapies likely reflects a broader mechanistic challenge. Most available agents were developed for generalized neuropathic pain syndromes rather than treatment-induced neurotoxicity and therefore inadequately address the mitochondrial, neuroimmune, and nociceptive mechanisms implicated in CIPN [10,16,28,65]. In addition, substantial biologic heterogeneity across chemotherapy classes and patient populations suggests that a uniform treatment paradigm may be inherently insufficient.

Timing may represent an additional therapeutic limitation. Many interventions are introduced only after neuropathy becomes clinically established, potentially after maladaptive neuroplastic and neuroimmune processes have become self-sustaining. This may help explain why symptomatic improvement is often incomplete even when treatment is initiated promptly after diagnosis.

Together, these limitations highlight the need for mechanism-informed and potentially disease-modifying therapeutic strategies rather than reliance on symptom-directed pharmacologic palliation alone.

### 5.2. Non-Pharmacologic and Supportive Interventions

Given the limited efficacy of pharmacologic therapies, increasing attention has been directed toward non-pharmacologic and supportive interventions for CIPN. These approaches may improve symptoms while avoiding many of the adverse effects associated with neuropathic pain medications. However, evidence supporting most interventions remains mixed, and standardized treatment protocols are lacking.

Exercise-based interventions have emerged as one of the more promising supportive strategies. Aerobic exercise, resistance training, and balance-focused rehabilitation programs have demonstrated potential benefits in symptom severity, functional impairment, balance, and quality of life among patients with CIPN [67,68,69]. Exercise may also influence inflammation, mitochondrial function, and neuroplasticity, although the precise mechanisms remain incompletely understood [67,68,69]. Benefits appear greatest when programs are individualized and initiated before severe functional decline develops.

Cryotherapy and compression therapy have received increasing attention, particularly as preventive strategies during taxane administration. Limb cooling may reduce drug delivery to peripheral nerves through localized vasoconstriction and thereby decrease neurotoxicity [70,71,72,73]. However, variability in study design and treatment protocols limits definitive conclusions regarding efficacy and implementation.

Acupuncture has also shown potential benefit in selected patients with painful CIPN, with some studies reporting improvements in pain, sensory symptoms, and quality of life [74,75]. Proposed mechanisms include modulation of endogenous opioid signaling, neuroimmune pathways, and central pain processing [74]. Neuromodulatory approaches, including scrambler therapy, transcutaneous electrical nerve stimulation (TENS), and other neurostimulation techniques, have likewise generated interest as adjunctive therapies for refractory CIPN [76,77]. While early findings are encouraging, larger randomized studies are needed to determine durability of benefit and optimal patient selection.

Importantly, supportive management extends beyond symptom reduction alone. Physical therapy, occupational therapy, gait stabilization, fall prevention strategies, psychosocial support, and survivorship-focused multidisciplinary care may substantially influence long-term functional outcomes and quality of life, even when complete symptom resolution is not achievable [11,78,79].

Overall, multimodal supportive care may provide meaningful benefit for selected patients with CIPN, although further mechanistically-informed studies are needed to identify which patients are most likely to benefit from specific interventions.

From a clinical perspective, early recognition of persistent CIPN, routine assessment of symptom severity and functional impairment, and timely implementation of supportive interventions remain essential for optimizing long-term outcomes. Given the limited efficacy of currently available pharmacologic therapies, multidisciplinary survivorship remains the cornerstone of clinical management.

Current management strategies and emerging mechanism-directed therapeutic approaches are summarized in Table 2.

## 6. Future Directions and Emerging Therapeutic Paradigms

Recent advances in the understanding of CIPN biology have created opportunities to move beyond traditional symptom-based management paradigms. Emerging biomarkers, improved mechanistic characterization, and growing recognition of survivorship-related morbidity suggest that future care models may be increasingly individualized, mechanism-informed, and focused on prevention of chronic pain persistence. These developments may ultimately reshape both therapeutic development and long-term management strategies for patients with CIPN.

### 6.1. From Uniform Toxicity Models to Biologically Stratified CIPN

A major emerging challenge in CIPN research is the recognition that the syndrome likely represents a biologically heterogeneous chronic neuropathic pain syndrome rather than a single mechanistically uniform toxicity. Although CIPN is commonly classified according to symptom severity or chemotherapy exposure, growing evidence suggests that distinct biologic processes—including mitochondrial dysfunction, neuroimmune activation, dorsal root ganglion toxicity, ion channel dysregulation, and central sensitization—may contribute differently across chemotherapy classes and individual patients [17,18,19,20,21,22,23,24,53,54,55,56,57,58,59,60,61,62].

Historically, most CIPN treatment strategies have relied on generalized neuropathic pain paradigms in which biologically diverse patients are managed using a largely uniform symptom-based framework. This may partially explain the inconsistent efficacy observed across many pharmacologic and non-pharmacologic interventions despite strong mechanistic rationale [6,16,28,60,64].

Going forward, therapeutic development may therefore depend on mechanism-informed and biologically individualized approaches. Emerging biomarkers such as neurofilament light chain, circulating mitochondrial DNA, inflammatory cytokine profiles, glial activation markers, and microRNA signatures may eventually support mechanistic phenotyping, risk stratification, and individualized therapeutic selection [30,31,34,40,53]. Such approaches could help identify patients at greatest risk for persistent CIPN and guide earlier intervention before maladaptive neuroplastic and neuroimmune changes become established.

**Figure 2:**
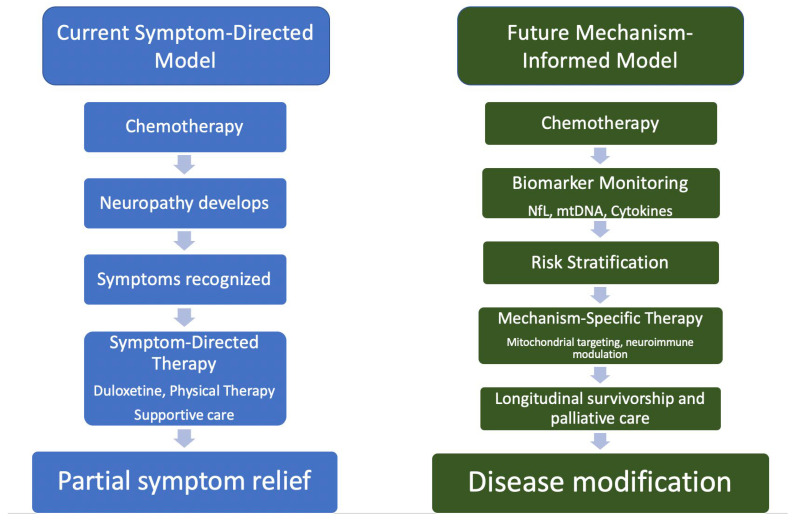
Current versus future care paradigms for CIPN. Current management strategies typically recognize CIPN after symptom development and focus on symptomatic treatment. Future approaches may incorporate biomarker surveillance, biologic stratification, mechanism-specific intervention, and longitudinal survivorship and palliative care to reduce chronic pain persistence and functional impairment.

This transition reflects a broader shift from reactive symptom management toward biomarker-informed risk stratification and mechanism-based intervention (Figure 2).

Potential clinical trials may increasingly require biomarker-enriched enrollment strategies, chemotherapy-specific therapeutic approaches, and longitudinal biologic monitoring to identify differential responses across distinct neuropathy phenotypes [16,28,60,64]. As disease-modifying therapies emerge, biologic stratification may become increasingly important for matching patients to the most appropriate interventions.

### 6.2. Rethinking Therapeutic Development and Longitudinal Care in CIPN

A central limitation of contemporary CIPN management is that therapeutic development has historically remained poorly aligned with the chronic and multidimensional nature of the syndrome. As a result, many interventions that provide modest symptomatic benefit have shown limited ability to meaningfully improve long-term disability, functional decline, or quality of life.

This limitation is particularly important because persistent CIPN frequently extends far beyond active cancer treatment. Many patients continue to experience chronic neuropathic pain, impaired mobility, gait instability, sleep disturbance, and reduced physical function months or years after chemotherapy completion. Yet relatively few therapeutic studies incorporate longitudinal follow-up capable of capturing survivorship-related outcomes. The next generation of clinical trials may therefore require greater emphasis on functional mobility, balance, independence, sleep quality, return to work, and overall quality of life rather than pain severity alone.

Increasing recognition of persistent CIPN as a survivorship and cancer pain issue also suggests that future management must extend beyond conventional toxicity-monitoring paradigms. Patients frequently transition among oncology, neurology, rehabilitation, pain medicine, survivorship, and palliative care settings without a clearly integrated long-term management strategy. More comprehensive multidisciplinary models incorporating rehabilitation, psychosocial support, chronic pain management, and survivorship-focused care may therefore become increasingly important.

An important next step in CIPN management may depend not only on development of new therapeutic agents, but also on broader changes in how persistent CIPN is monitored, studied, and managed across the continuum of cancer care. Greater emphasis on longitudinal outcomes, multidisciplinary survivorship care, functional preservation, and chronic pain-oriented treatment frameworks may ultimately prove essential for improving quality of life and long-term recovery in patients living with persistent CIPN.

### 6.3. Key Clinical Messages

- Persistent CIPN should be recognized as a chronic neuropathic syndrome rather than solely a chemotherapy-related toxicity.
- Early recognition and chemotherapy dose modification remain the most effective strategies for limiting long-term neurologic toxicity.
- Duloxetine remains the only ASCO-guideline support pharmacologic therapy for established painful CIPN
- Multidisciplinary survivorship care, including rehabilitation and fall prevention, is essential for optimizing long-term functional outcomes.
- Emerging mechanism-informed and disease-modifying therapies remain investigational but represent promising future therapeutic directions.

## 7. Conclusions

CIPN remains one of the most common and therapeutically challenging complications of modern cancer treatment. Although traditionally viewed as a chemotherapy-related toxicity, growing evidence suggests that persistent CIPN more closely resembles a biologically heterogeneous chronic neuropathic cancer pain syndrome characterized by mitochondrial dysfunction, neuroimmune activation, maladaptive sensitization, and impaired neuronal recovery. This framework helps explain the limited efficacy of many current symptom-directed therapies while providing a rationale for mechanism-informed therapeutic development.

As cancer survivorship continues to improve, the long-term burden of persistent CIPN is becoming increasingly consequential. Future progress will likely require earlier intervention, mechanism-informed therapeutic development, biologic stratification, and survivorship-focused multidisciplinary care. Reframing persistent CIPN as a chronic cancer pain syndrome is not merely a conceptual shift—it is essential to advancing mechanism-informed therapeutics, multidisciplinary survivorship care, and long-term outcomes for patients living with chronic neuropathic pain after cancer treatment.

## Figures and Tables

**Table 1:** Major Neurotoxic Chemotherapy Classes and Clinical Characteristics of CIPN.

Chemotherapy Class	Representative Agents	Predominant Mechanism	Clinical Diagnosis	Common Symptoms	Predominant Neuropathy Type
Taxanes	Paclitaxel, docetaxel	Microtubule stabilization; impaired axonal transport	Sensory neuropathy	Acute pain syndrome; numbness; paresthesias	Painful/Sensory (P/S)
Platinum agents	Oxaliplatin, cisplatin, carboplatin	Dorsal root ganglion toxicity; DNA damage; oxidative stress	Sensory neuropathy	Cold hypersensitivity; paresthesias; neuropathic pain	Painful/Sensory (P/S)
Vinca alkaloids	Vincristine, vinblastine	Microtubule disruption and impaired axonal transport	Sensorimotor neuropathy	Weakness; numbness; gait instability	Motor Sensory (M/S)
Proteasome inhibitors	Bortezomib, carfilzomib	Mitochondrial dysfunction, neuroinflammation	Painful sensory neuropathy	Burning pain; allodynia	Painful (P)
Immunomodulatory agents	Thalidomide, lenalidomide	Axonal degeneration	Sensory neuropathy	Numbness; gait instability	Sensory (S)

Abbreviations: CIPN, chemotherapy-induced peripheral neuropathy; Data summarized from references [58,59,60,61] and related studies discussed in the text.

**Table 2:** Current and Emerging Therapeutic Strategies for CIPN.

Therapeutic Strategy	Examples	Rationale	Clinical Status/Limitation
Guideline-supported pharmacologic therapy	Duloxetine	Descending serotonergic/noradrenergic pain modulation	Recommended for painful CIPN; benefit is modest and variable
Other neuropathic pain agents	Gabapentin, pregabalin, tricyclic antidepressants	Symptomatic neuropathic pain modulation	Frequently used; inconsistent CIPN-specific efficacy
Exercise and rehabilitation	Aerobic exercise, resistance training, balance training, PT/OT	Functional restoration, neuroplasticity, fall prevention	Growing supportive evidence; protocols remain heterogeneous
Preventive physical strategies	Cryotherapy, compression therapy	Reduced peripheral drug exposure during chemotherapy	Emerging prevention strategy; variable protocols and mixed evidence
Integrative/neuromodulatory therapies	Acupuncture, scrambler therapy, TENS	Peripheral and central pain modulation	Adjunctive/investigational; larger trials needed
Mitochondrial-targeted approaches	ALT001, salidroside	Mitophagy induction and mitochondrial quality control	Preclinical; not clinically validated
Neuroimmune/biomarker-guided approaches	Fucoidan, Gas6/MerTK targeting, NfL, mtDNA, cytokines, microRNAs	Neuroimmune modulation, risk stratification, biologic phenotyping	Translational/emerging; requires human validation

Abbreviations: CIPN, chemotherapy-induced peripheral neuropathy; mtDNA, mitochondrial DNA; NfL, neurofilament light chain; PT, physical therapy; OT, occupational therapy; TENS, transcutaneous electrical nerve stimulation.

## Data Availability

No new data were created or analyzed in this study. Data sharing is not applicable to this article.
